# Genetic Structure of Cultivated Varieties of Radicchio (*Cichorium intybus* L.): A Comparison between F1 Hybrids and Synthetics

**DOI:** 10.3390/plants8070213

**Published:** 2019-07-10

**Authors:** Alice Patella, Francesco Scariolo, Fabio Palumbo, Gianni Barcaccia

**Affiliations:** Department of Agronomy, Food, Natural Resources, Animals and Environment, University of Padova, 35020 Legnaro PD, Italy

**Keywords:** SSR markers, red-chicory, genotyping, microsatellite, DUS test, plant breeders’ rights

## Abstract

*Cichorium intybus* L., well known in Italy with the common name “Radicchio”, is an important leafy vegetable that is prevalently reproduced by allogamy due to very efficient barriers of self-incompatibility. Marker-assisted breeding is widely used by seed firms to develop new hybrid varieties that manifest genetic distinctiveness, uniformity and stability. A total of 29 mapped microsatellite markers were used for genotyping 504 samples of the Red of Chioggia biotype: First, two synthetics, four F1 hybrids and two derived F2 populations were compared to assess the distinctiveness of their gene pool and structure; then, the uniformity and stability of 3 years of production of a commercial F1 variety were also investigated. Genetic similarity and diversity statistics as well as the genetic structure of populations were analysed, including allele and genotype frequencies. The mean estimates and ranges of genetic similarity enabled the molecular discrimination of OP synthetics from F1 varieties and their F2 progenies and the determination of individual plant memberships. Moreover, the genetic structure of F1 hybrids produced in 3 years unexpectedly revealed two main clusters that discriminate the first 2 years from the 3rd, mainly because of the presence of uncommon specific alleles and different allele frequencies. Overall, this molecular information will enable breeders to determine the genetic distinctness, uniformity and stability of commercial and experimental varieties, as well as their genetic relationships and relatedness. Hence, this work provides a useful tool for achieving the molecular characterisation and genetic identification of different radicchio populations.

## 1. Introduction

Radicchio (*Cichorium intybus* subsp. *intybus* var. *foliosum* L., 2*n* = 2*x* = 18) is the Italian name of an important locally-cultivated leaf chicory belonging to the Asteraceae, one of the largest families among flowering plants. From a reproductive perspective, radicchio is prevalently allogamous due to an efficient sporophytic self-incompatibility system and presents entomophilous pollination [1]. Moreover, outcrossing is promoted by a floral morpho-phenology that creates a physical barrier to self-pollination in the absence of pollen donors and favourable competition of allo-pollen grains and tubes [2].

Among the different biotypes available in radicchio, Red of Chioggia is one of the most commercially relevant. Historically, commercial varieties were developed by recurrent mass selection, but in recent years, synthetics have been constituted by breeders through inter-crossing or poly-crossing a number of mother individuals or clonal lines selected on the basis of their morpho-phenological and agronomic traits and, eventually, by performing progeny tests to assess their general combining ability [3]. Currently, owing to the economic benefits, newly released varieties are mainly F1 hybrids developed by Italian or European seed firms through large-scale single crosses between inbred lines selected according to their specific combining ability and exploiting molecular marker-assisted breeding (MAB) strategies. Thus, radicchio breeding programmes have improved significantly in recent years due to more efficient biotechnological tools [4].

In this regard, several linkage maps saturated with DNA markers and spanning the entire genome size (approximately 2.6 Gb) are available for leaf chicory [5,6,7,8,9]. These maps are particularly relevant considering that biotechnology and molecular genetics are largely utilised in programmes for breeding radicchio [10], as well as the vast majority of crop plant species [11]. In this context, the linkage map developed by Cadalen et al. [5] in chicory (*C. intybus*) is of particular interest. This genetic map is based on 431 SSR and 41 STS markers and includes nine linkage groups obtained after the integration and organisation of molecular marker data derived from one witloof chicory and two industrial chicory progenies [12]. Among codominant molecular markers, SNPs are advantageous for their abundance and high frequency in the genome and for their efficiency, but most SNPs are limited by their biallelic nature. In contrast, SSR markers are characterised by multiallelism, a mostly single-locus inheritance with a relatively lower cost. Moreover, a robust and reliable genotyping method based on SSR markers is already available for radicchio [10]. In this study, we present the implementation of the research of Ghedina et al. [10], who identified an efficient method for assessing a multi-locus genotype of plant individuals and lineages aimed at the selection of new varieties and the certification of local firm products. Our research includes the implementation of a DNA genotyping method useful for assessing the genetic distinctness and population structure of various commercial open pollinated (OP) synthetics, F1 hybrids and their F2 progenies belonging to the biotype Red of Chioggia. In addition, our research addresses the molecular characterisation and comparison of an F1 hybrid variety produced in 3 years (2014, 2015 and 2016) and obtained by open field crossings. The aim consists of evaluating the genetic uniformity and stability of these plants in different commercial lots, exploiting the same set of markers used for genotyping the other samples.

## 2. Results

### 2.1. Genetic Structure of Commercial Open Pollinated (OP) Varieties, F1 Hybrids and their F2 Progenies

A total of 216 samples belonging to two synthetics lines (OP-1 and OP-2), four F1 hybrids (F1-A, F1-B, F1-C, F1-D) and two F2 progenies (F2-C and F2-D produced from F1-C and F1-D, respectively) were investigated with 29 SSR markers. Samples were also chosen among short and medium–long term development cycle materials to equally represent the Chioggia biotypes currently available on the market (Table 1).

All SSR markers were polymorphic, and the mean polymorphic information content (PIC) value was 0.61, with a maximum equal to 0.84 for the M2.6 SSR locus. Of note, 24 of 29 SSR markers were highly informative [13], with PIC values higher than 0.5. Two other loci were considered informative (M5.15 and M7.21, 0.25 < PIC < 0.5), while M3.7, M8.22 and M5.14 were less informative (PIC < 0.20, Supplementary Table S1). The total number of scored alleles was 220, with 2.7 observed alleles (Na) per locus and 2.0 expected alleles (Ne) per locus (Supplementary Table S1). The mean Na for a single locus was higher in synthetics (4.5 alleles/locus) than in F1 hybrids (2.3 alleles/locus) and F2 progenies (2.8 alleles/locus).

Additionally, the mean Ne was higher in the OP populations (2.6 alleles/locus) than in the F1 hybrids and F2 progenies (1.8 and 1.9 alleles/locus, respectively, Table 2). Private alleles were observed in 25 of 29 SSR loci, but in only 11 of these SSR loci was the allele frequency higher than 15%. Specifically, the F1-B hybrid had private alleles in four loci with frequencies >15.0% (M2.4 50.0%, M1.1 40.0%, M4.12 and M6.17 15.0%); instead, the F1-C hybrid had one locus showing a private allele with a 25.0% frequency (M6.17). In the case of OP-1, two private alleles were detected at two different loci with frequencies >30.0% (M6.17 at 50.0% and M6.18 at 31.0%) and five other loci with frequencies >15.0% (Supplementary Table S2). The locus with the highest number of private alleles across the population was M6.17, although it did not represent the locus with the higher PIC value, being equal to 0.7 (Supplementary Table S1).

Allele frequencies were calculated per locus within each population, permitting the identification of the most common genotype. In contrast to synthetics, within the hybrid pool, a fixed genotype was found across several loci (Supplementary Tables S3 and S4). Specifically, we found a mean of 8.8 fixed genotypes for hybrids and only 3.0 for synthetics.

**Figure 1:**
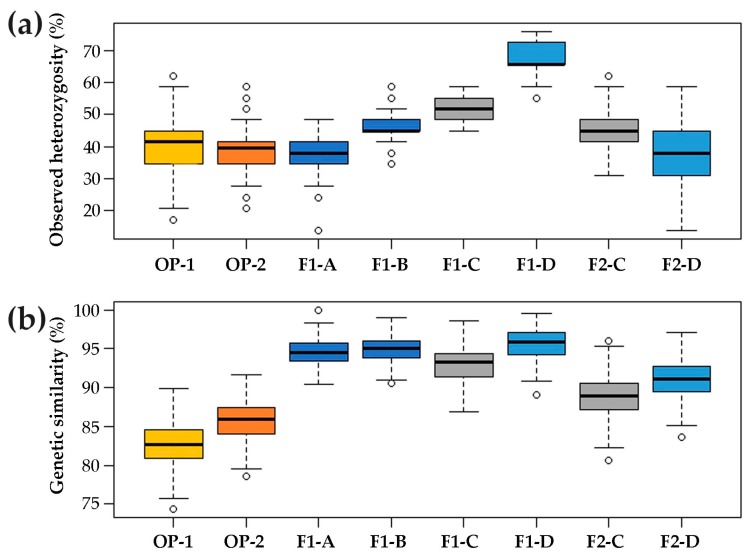
Statistics of genetic diversity and similarity for OP synthetics, F1 hybrids and F2 progenies. (**a**) Box plot of the observed heterozygosity within each population. (**b**) Box plot of the median genetic similarity (MGS) within each ID (in percentage). The second and third quartiles are marked inside the square and are divided by a bold bar (median). Dots show outlier samples.

The observed heterozygosity (Ho) of F1 hybrids, considered as a whole, was 50.0% on average (ranging from 37.9% to 65.5%, Figure 1a), and in particular, F1-C and F1-D exhibited the median highest values (51.7% and 65.5%, respectively). The two OP synthetics showed a median heterozygosity as low as 40.5% (from 39.7% to 41.4%). Moreover, the median percentage of heterozygosity within the two F2 progenies (F2-D and F2-C) was 41.4% (from 37.9% to 44.8%), considerably lower than the one calculated for the two F1 hybrids used as parents (F1-C and F1-D) (Figure 1a). The median observed heterozygosity of F1-A, and F1-B resulted in 37.9% and 44.8%, respectively (Figure 1a). Additionally, the expected heterozygosity (He) was lower than the observed heterozygosity in the F1 populations and F2-C (Supplementary Table S5).

According to the similarity analysis conducted using NTSYS software, the median estimate of genetic similarity within each population was higher for hybrid varieties (95.2% on average, ranging from 94.0% to 97.1%) than synthetics (84.3%). In the case of F2 progenies, the genetic similarity within each population was 89.1% and 90.7% for F2-C and F2-D, respectively (Figure 1b). The mean genetic similarity (MGS) calculated among F1 hybrids ranged from 76.4 ± 1.5% (F1-B vs. F1-D) to 87.2 ± 1.6% (F1-A vs. F1-B), while those calculated between F1-C vs. F2-C and F1-D vs. F2-D were both slightly over 90% (90.2 ± 2.5%). Moreover, F1-A and F1-B were highly similar to each other (87.2 ± 1.6%) (Supplementary Figure S1).

An unweighted pair group method with arithmetic mean (UPGMA) dendrogram was also constructed on the basis of the genetic similarity matrix, whose coefficients were computed in all possible pair-wise combinations of the 216 samples. The dendrogram clustered the entire collection in six main subgroups. Each cluster, with few exceptions, included the whole pool of samples used to represent each of the six commercial lines. Additionally, as shown by the genetic similarity matrix, OP-1 resulted in the most dissimilar group compared to the rest of the samples, and the corresponding branch of the dendrogram was clearly separated from the rest of the tree. Notably, the UPGMA did not enable the full discrimination of the F2 progenies that grouped together with their respective F1 parents (Supplementary Figure S1).

**Figure 2:**
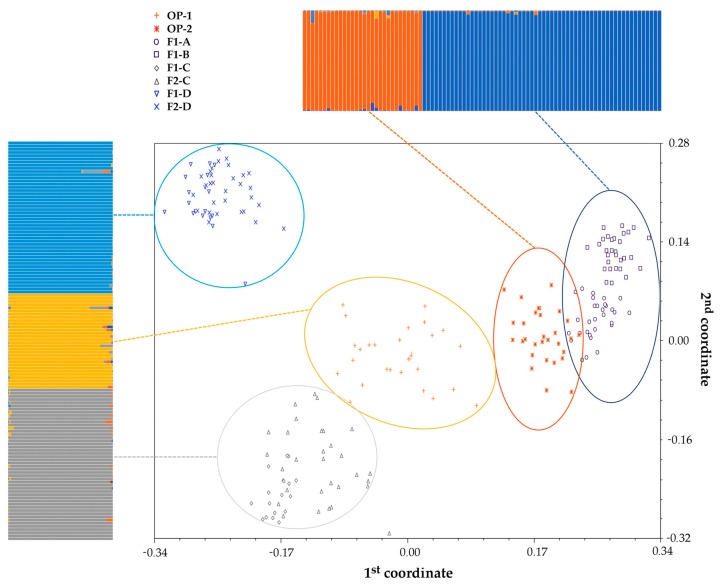
Principal coordinate analysis (PCoA). The centroids of all radicchio samples (*n* = 216) deriving from the mean genetic similarity coefficients plotted according to the first two main coordinates. For each population, the PCoA output is coupled with the results of the population genetic structure study, estimated by STRUCTURE software using the same SSR marker data set. Each sample is represented by a vertical histogram partitioned into *K* = 5 coloured segments that report the estimated membership.

According to the principal coordinate analysis (PCoA), similar to what was highlighted by the UPGMA tree, the two synthetic populations clustered independently from the rest of the groups, although a partial overlap was observed between OP-2 and F1-A. In contrast, F1-C and F1-D formed unique clusters with their respective F2 progenies. The first principal coordinate accounted for 8% of the total variation and clearly separated a group including F1-A, F1-B and OP-2 from the group of F1-C, F2-C, F1-D, and F2-D; the second principal coordinate accounted for 5% of the total variation and separated F1-C and F2-C from F1-D and F2-D. Finally, the hybrid varieties F1-A and F1-B were highly similar genetically, forming a cluster divided into two subgroups (Figure 2).

Regarding the investigation of the genetic structure of the radicchio core collection, the best estimate of population size was *K* = 5 such that the 216 samples were grouped into five genetically distinct clusters. Each genotype was plotted as a vertical histogram divided into *K* = 5 coloured segments representing the estimated membership in each hypothesised ancestral genotype. A total of 212 of 216 individuals showed strong ancestry association (>90.0%), and two samples scored a slightly lower level of association (equal to 87% and 88%, respectively); only two additional samples had a very low degree of ancestry association (75.0% and 70.0%, respectively), and they were hence considered admixed. Moreover, the two F2 progenies were not distinguished from their original hybrids, and F1-A and F1-B hybrids were grouped as if they had only one common ancestor (Figure 2), analogous to what was observed in the PCoA.

### 2.2. Genomic Comparison among Three Years Production of An F1 Hybrid Variety

Regarding the genomic comparison of the 3 production years of commercial lots of an F1 hybrid (2014, 2015 and 2016), the level of genetic differentiation between samples was investigated by calculating the genetic similarity in all possible pair-wise comparisons among all 288 individual plants (96 samples per year).

The Rohlf’s coefficient of genetic similarity ranged from 65.0% to 100%, with an estimated average equal to 91%. Additionally, a comparison within and between the 3-year populations was performed to assess the stability of the F1 hybrid variety. Regarding the year 2014, the median genetic similarity observed was 91.8%, ranging from 66.4% to 100%; 2015 went from 77.0% to 100% with a median genetic similarity of 95.4%, while 2016 showed a median genetic similarity equal to 89.7%, ranging within the population from 80.0% to 100%. Moreover, comparisons among the different populations were made. The median values were 92.0%, 89.0% and 90.8% for the 2014 vs. 2015, 2014 vs. 2016 and 2015 vs. 2016 comparisons, respectively. For the minimum genetic similarity data, the lowest value was observed in 2014 vs. 2016 (65.4%), followed by 2014 vs. 2015 (67.0%), and the highest value was in 2015 vs. 2016 (75.2%).

**Figure 3:**
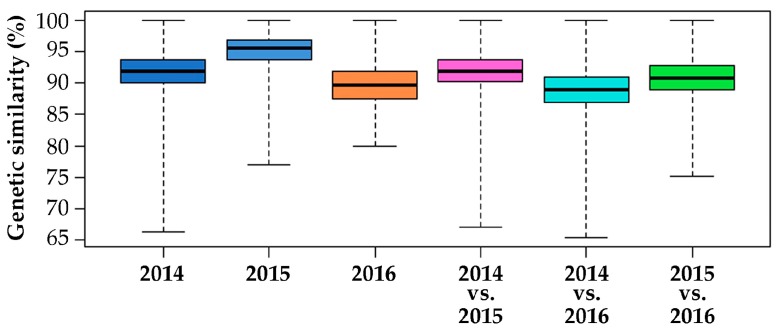
Box plot of the genetic similarity of 3 years of production of an F1 hybrid variety, calculated within and among individuals of years 2014, 2015 and 2016. The second and third quartiles are marked inside the square and divided by a bold bar (median).

Figure 3 shows the genetic similarity statistics for the 3 years of production of the analysed F1 hybrid variety, calculated within and among individuals of years 2014, 2015 and 2016.

**Figure 4:**
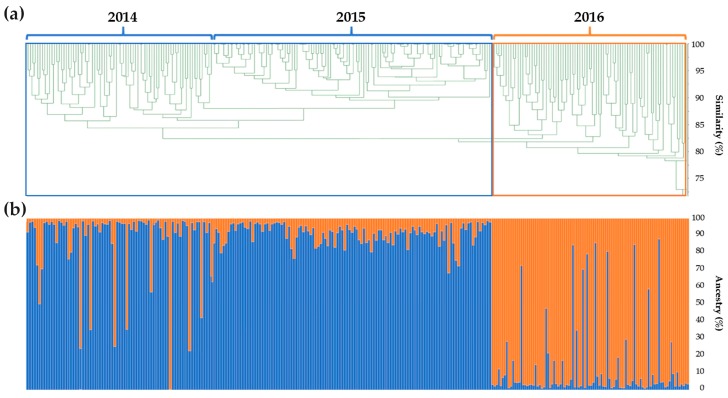
Analysis of the genetic structure of the 3 years of seed production of the F1 hybrid variety analyzed. (**a**) Unweighted pair group method with arithmetic mean (UPGMA) dendrogram, based on the calculated genetic similarity among individuals of years 2014, 2015 and 2016. Blue and orange squares highlight the two main identified clusters. (**b**) STRUCTURE software results. Data are disposed of in a vertical histogram, labelled for *K* = 2 colours concerning cluster membership of each individual sample belonging to years 2014, 2015 or 2016.

With the data obtained from the genetic similarity analysis, a UPGMA dendrogram was also computed, highlighting the clustering in two main subgroups. The first group included individuals belonging to both years 2014 and 2015, while the second group, strongly divided from the previous years, prevalently clustered 2016 plants (Figure 4a).

From the analysis of the genetic structure of the 3 years of seed production, the most likely value of K was 2 for the population as a whole. Each genotype of the analysed F1 hybrid variety was plotted in a vertical histogram divided into *K* = 2 coloured segments representing the estimated membership in each hypothesised ancestral (Figure 4b). From this analysis, reflecting the results obtained from the UPGMA dendrogram, almost all of the 2014 and 2015 samples grouped in cluster 1, while 2016 individuals grouped in cluster 2. Clustering revealed that 246 of 288 samples (85.4% of samples) showed a strong ancestry association (>85.0%). Twenty-three admixed samples were part of the first 2 years of production (2014 and 2015, cluster 1), while the remaining 19 admixed genotypes belonged to the 2016 production (cluster 2). It is relevant to note that few samples scored a membership perfectly fitting the second cluster with matching values over 99%. At the same time, relative to the year 2016, which was mainly grouped in cluster 2, some admixed samples showed very low membership values.

**Figure 5:**
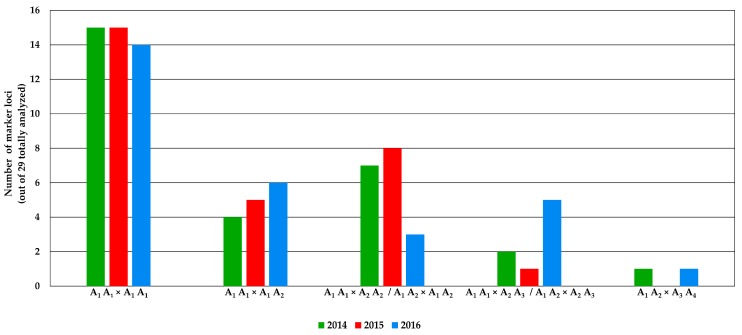
Number of loci corresponding to the five allelic frequency profiles derived from hypothetical crosses between representative genotypes in the 3 different analysed years (2014, 2015 and 2016).

From the analysis of the allele frequencies, it emerged that for 2014 and 2015, 51.7% of loci (15 out of 29 loci) matched the hypothetical profile by which both parental genotypes used for crossing are homozygous for the same allele, while for 2016, 48.7% of loci (14 out of 29 loci) matched. Regarding the second putative profile (A_1_A_1_×A_1_A_2_), 13.8%, 17.2% and 20.7% of loci (4, 5 and 6 out of 29 loci, respectively) matched for the years 2014, 2015 and 2016, respectively. The year that best represented the third case (A_1_A_1_×A_2_A_2_/A_1_A_2_×A_1_A_2_) was 2015 (27.6% of loci, i.e., 8 out of 29 loci) followed by 2014 (24.1%, i.e., 7 out of 29 loci) and 2016 (10.3%, i.e., 3 out of 29 loci). In contrast, 2016 matched the fourth hypothesis (A_1_A_1_×A_2_A_3_/A_1_A_1_×A_1_A_3_) with a percentage equal to 17.2%, which is representatively higher than for 2014 and 2015, and fits 6.9% (2 out of 29 loci) and 3.4% (1 out of 29 loci), respectively. Regarding the last cluster of the bar chart (A_1_A_2_×A_3_A_4_), where both parental genotypes are heterozygous for different alleles, 3.4% of the loci showed correspondence for 2014 and 2016, while there were none for 2015 (Figure 5).

Considering the allele frequencies, it is noteworthy that five loci (M1.2, M1.3, M2.6, M8.24 and M9.25) showed new alleles in 2016, with rates extending from 8.6% (M1.3) to 15.5% (M1.2) (Table S6a). Moreover, in 2014, at locus M9.26, allele A_1_ was present, but it disappeared in 2015, and in 2016, it was replaced by a new allelic variant (allele A_3_). In contrast, two polymorphisms at the M8.23 (allele A_7_) and M9.27 (allele A_6_) loci were detected in 2014 and 2016 but not in 2015 (Table S6b). Another result emerging from the allele frequencies analysis is that there were polymorphisms at many loci (82.8%) among all 3 years, with percentages under 3.0% being observed. These results were not considered during the analysis because they are probably derived from pollen contamination.

## 3. Discussion

Traditional methods have recently been integrated with biotechnological methods to accelerate breeding programmes. Marker-assisted breeding (MAB), in fact, is widely utilised for the development of improved lines by firms and research institutes, allowing breeding activity based not only on the evaluation of phenotypes but also on plant genotypes. Moreover, molecular assays have become useful tools for verifying the distinctness, uniformity and stability of varieties (DUS test), three major requirements for the registration of new plant materials. Microsatellite markers can identify essentially-derived varieties (EDV) in the context of variety registration; therefore, these markers represent useful tools for cultivar protection against plagiarism. [14]. In particular, molecular assays based on SSR markers overcome the mostly subjective system of morpho-phenological characterisation. Our genotyping investigation of the radicchio cultivated lines of the Chioggia biotype fits well with this scenario. Specifically, the present study enabled the comparison of different commercial varieties of high breeding value by improving the method of Ghedina et al. [10] based on SSR markers. Two loci were added to the original number (27), and the multiplex PCRs were reduced from 4 to 3. The 29 SSR molecular markers used in this work were chosen because they were equally distributed throughout the whole genome and dispersed over the nine LGs (minimum of three markers for each LG), making the molecular assay efficient. Moreover, this method was shown to be an inexpensive and fast tool for genotyping analyses. This panel of SSR markers was first used to evaluate the genetic variation within and among a core collection of commercial OP synthetics, F1 hybrids and their F2 progenies. Moreover, the same set of microsatellites was also used to investigate the genetic structure and genetic similarity of 3 production years of an F1 hybrid variety. Additionally, this study provides useful tools for protecting registered varieties against plagiarism.

### 3.1. Genetic Structure of A Core Collection of OP, F1 and F2 Populations

Marker tools differ in their information content, depending on their polymorphism degree. The PIC calculated for the 29 SSR marker loci showed an average value of 0.61; therefore, the microsatellite markers used in this study were determined to be generally highly informative. According to Botstein et al. [13], 24 of the selected SSR markers could be considered highly informative (PIC > 0.5), while only five SSR markers were considered less informative. The highest Na and Ne values (Na = 4.5, Ne = 2.6), observed within the two OP synthetics, directly correlate with the population size and with a high genetic diversity, which was later found within them both. In fact, a high Na should produce many genetically possible genotypes and thus low genetic similarity within the population. In contrast, this property was not found for the F1 progenies. Considering the F1 generation as a whole (i.e., 96 samples), the population size was larger than that of the OP synthetics, but both Na and Ne were significantly lower (2.3 and 1.8, respectively) (Table 2). However, this finding is consistent with the progenies’ breeding history and with the high similarity highlighted within the F1 populations.

A high number of private alleles were detected within the synthetic populations, especially in OP-1, where private allelic variants were found at seven SSR loci; notably, in two cases, the frequency was > 30%. Interestingly, private alleles with high frequencies were also identified in F1-B and F1-C, making them attractive tools for protecting the rights of plant breeders [15]. Additionally, a fixed genotype across several loci of the hybrid varieties was found. Specifically, we observed a mean of 8.8 fixed genotypes for hybrids and a mean of 3.0 for synthetics. Thus, it is reasonable to think that a combination of those SSR loci exhibiting private alleles could be profitably exploited to protect and trace registered varieties, as well as their derivative food products. High uniformity and the ability to trace hybrid products explains the high exploitation of F1 seeds. Indeed, since these varieties have the same genotypes, farmers adopted hybrids for combining such qualities as maturation contemporaneity and productivity traits.

The significant variability, in terms of heterosis, shown by the four F1 hybrid populations, relies on the genetic distance between the relative parents. In fact, information derived from genotyping data are exploited more and more for planning crosses and predicting plant vigour traits (i.e., heterosis) of experimental F1 hybrids on the basis of the overall genetic distance and allelic divergence between parental inbred lines, as an estimate of their specific combining ability [10,16]. As occurs for most open-pollinated species, detectable heterotic effects are well-known also in Radicchio [16,17]. In this species, it has been demonstrated that hybridization between selected genotypes provides uniform and heterotic populations due to increased heterozygosity: Field trials performed using commercial F1 hybrids showed that the genetic diversity between paternal and maternal lines is positively correlated not only with the observed degree of heterozygosity of their hybrid progenies, but also with the realized crop yield potentials of individual plants. In particular, F1 hybrids of Radicchio can manifest an increase of leaf yields per single plant equal to 25–30%, on average, if compared to OP synthetics of the same varietal cycle length (unpublished data, Blumen Group SpA). This is why recently private breeders and seed firms have implemented methods for the development of F1 hybrids [8]. Therefore, we can speculate that those F1 offspring that showed lower heterozygosity (i.e., F1-A and F1-B with 37.9% and 44.8%, respectively) are the results of crosses between parents that are homozygous for the same alleles at the considered loci. As expected, both the OP synthetics and the F2 progenies showed a lower heterozygosity compared to the F1 hybrids, F1-A excluded. However, the reduction in heterozygosity observed in the F2 populations is the direct consequence of segregation events, while the low levels found in the OP synthetics are the result of a long breeding process aiming to constitute highly uniform populations. Notably, the genetic similarity calculated for F1-C and F1-D is shown to be higher than those reported in their direct F2 progenies. The reduction in the genetic similarity values of the F2 populations can still be attributed to the segregation events that cause progenies to be less similar within them but with an increment of their genetic similarity range. The median estimate of genetic similarity within populations was higher in hybrid (95.2%) than in synthetic (84.3%) varieties, which was in full agreement with the breeding strategies exploited for their development. This result explains and gives rise to the greater stability that is usually appreciated in the hybrid cultivated varieties compared to the non-hybrids. Moreover, the genetic stability of a population also facilitates the employment of molecular data for breeders’ rights protection. On the one hand, it is feasible to identify and exploit private alleles for the unequivocal identification of commercial hybrids; on the other hand, the higher genetic variability found within the OP synthetics highlights the difficulty of protecting them from frauds.

Other information regarding the genetic similarity and the genetic structure of the analysed populations were provided by both the UPGMA dendrogram and the PCoA-based centroid, revealing well-separated sub-populations corresponding to the cultivated varieties object of this study. It is worth noting that the high similarity shown by the two hybrids F1-A and F1-B (median of genetic similarity equal to 87.2%) is also graphically evident in both analyses and is clearly the direct consequence of a common genetic root. The shared origin of these two hybrids is also visible from the STRUCTURE analysis, which confirms a common ancestor. The findings reported for F1-A and F1-B are also transposable to the two F2s (F2-C and F2-D) and their related F1 parents (F1-C and F1-D). The graphical overlapping in the PCoA and the high genetic similarity among the two generations are the expressions of a direct lineage, and their common progenitor further confirmed this finding. The only two samples considered admixed from the structure analysis can be assumed as off-types of these populations; thus, they will be removed from the core collection. Considering that a new variety, before the release on the market, needs to be distinct from all other commercially available cultivars, the results of this study indicated that SSR markers represent an efficient tool to evaluate the distinctness of commercial varieties or to investigate their common origin, as in the case of EDV.

### 3.2. Genomic Comparison among Three Different Production Years of A Commercial F1 Hybrid

From the genetic similarity analysis of the 3 years of hybrid seed production, samples belonging to 2015 showed the highest intra-similarity scores, with a median value of 95.4%, reflecting a generally higher uniformity of this population. In contrast, 2016 exhibited the lowest median similarity within the population, even being the year with the most contained variability among its individuals. At the same time, when considering the pairwise comparison between the different years, 2014 vs. 2015 appeared to be most similar, with a median value of 92.0%, followed by 2015 vs. 2016 (90.8%) and 2014 vs. 2016 (89.0%). Moreover, the 2015 vs. 2016 comparison showed the lowest variability, as the genetic similarity values ranged from 75.2% to 100% (Figure 3).

The genetic similarity data, also used for the construction of a UPGMA dendrogram, highlights a division in two main clusters. The first cluster consists of the years 2014 and 2015, while the other cluster consists of the year 2016. From the computed analysis, the dendrogram confirmed that two main clusters divide the year 2016 from the other two, 2014 and 2015, grouped together. It was also possible to observe that few samples were admixed (<85.0% of membership with their main group) among both clusters. Information was also confirmed by the UPGMA analysis, in which the highly different samples outstood the primary roots of the tree. To corroborate the clustering analyses, a comparison between observed allele frequencies at single loci per year and those obtainable in progenies after crossing different hypothetical parental genotypes was performed. Thus, five main patterns were supposed to categorise the different frequency profiles (e.g., A_1_A_1_×A_1_A_1_ gives p(A_1_) = 100%; A_1_A_1_×A_1_A_2_ gives p(A_1_) = 75% and q(A_2_) = 25%) among the 29 analysed loci over the 3 seed production years. After this step, all profiles across all loci were matched with the hypothetical profiles and then counted for each year. As described in Figure 5, most loci within each year (51.7%) showed having ancestors with a monomorphic genotype, but in 2016, this value decreased to 48.3%. In contrast, the second pattern (A_1_A_1_×A_1_A_2_) showed an average increase of 3.5% per year from 2014 to 2016, rising from 13.8% to 20.7%. This finding was considered to be an increase in the number of heterozygous loci in the parental lines used for the crossing. Additionally, the number of loci fitting the third and the fourth hypothetical profiles scored a substantial decrease in 2016 and increase in 2016, respectively (see Figure 5). This result is a further demonstration of the low genetic stability of the parental lines over the 3 years of production of the F1 hybrid. Another relevant result emerging from these data is that regarding the pattern obtained with crosses as A_1_A_1_×A_2_A_2_ and A_1_A_1_×A_2_A_3_ by which the resulting hybrid genotypes were observed in an overall frequency much lower than the one expected for F1 hybrid varieties. Notably, the fifth profile, characteristic of progenies obtained by crossing divergent heterozygous parental genotypes, was matched in years 2014 and 2016 for only 3.5% but not recorded in 2015. The investigation of unique alleles determined that in 2016, five different loci manifested having private alleles that were not found in the other two populations. Moreover, two alleles at different loci (M8.23 and M9.27) were present in 2014 and not 2015 and reappeared in 2016 with the same frequencies as in the first year, meaning that a correlation between the parental plants used for the constitution of this hybrid population is certain but with strong differences shown by the presence of specific alleles with elevated frequencies in the 2016 population. Interestingly, these results indicated the presence of a unique allele for marker M9.26 in 2014, substituted by an already existing polymorphism in 2015 and then replaced in 2016 by an entirely new polymorphism. These data enabled us to speculate that in 2014, parental genotypes were A_2_A_4_×A_1_A_2_; in 2015, they were A_2_A_4_×A_2_A_2_; and in 2016, they were A_2_A_4_×A_3_A_3_ (Supplementary Table S6).

Overall, these findings, from the genetic similarity comparison to the investigation of unique alleles, enabled a major consideration to explain the clustering and the poor genetic uniformity displayed by the 3 years’ productions. In particular, the fact that year 2016 clustered separately from the previous two can be interpreted as a strong contamination in the genetic pool of probably one parental line used in the crossing programme. Considering that both the parents and the F1 hybrid object of this study are grown in an open-field system without physical barriers; the high variability in terms of genetic similarity observed both within and among the 3 years of production could be consistent with some possible pollen contaminations, mainly from wild-type radicchio. This hypothesis is further corroborated by the presence of unique alleles (with frequencies under 3%), especially in 2016. Moreover, this second case study demonstrated the efficiency of microsatellite markers in assessing uniformity and stability through the generation of a commercial variety, two crucial requisites for its release and survival on the market.

## 4. Materials and Methods

### 4.1. Plant Materials and DNA Isolation

The plant material used in this study, including OP and F1 varieties, represents part of a high-breeding value collection of commercial lots belonging to the “Red of Chioggia” biotype of radicchio. In addition, F2 populations obtained ad hoc for the purpose of this investigation were also used.

In this study, 30 samples were used to represent each of the first two hybrids (F1-A and F1-B) and each of the two synthetic populations (OP-1 and OP-2); 36 samples were collected for F1-C (18) and F1-D (18) hybrids, and eventually, the two F2 progenies (F2-C and F2-D) were composed of 30 plants each and obtained by selfing single F1 individuals (respectively, F1-C and F1-D; Table 1). The F2s were specifically produced to evaluate their genetic structure compared to the synthetic genetic structure and to investigate the segregating pattern of some relevant F1 hybrids. The plant material was also chosen considering the varietal cycle: Two hybrids and a synthetic were characterised by a short cycle, while the remaining two hybrids and the second synthetic were distinguished for medium-long cycles (Table 1).

The second set of samples consisted of 288 plants belonging to three different populations obtained in 3 different years of production (2014, 2015 and 2016) from an F1 hybrid variety obtained in an open-field system without physical barriers.

On the whole, 504 samples were collected, and approximately 100 mg of fresh leaves were ground to fine powder using a TissueLyser II mill (Qiagen, Valencia, CA, USA). Genomic DNA was extracted with a DNeasy^®^ 96 Plant Kit (Qiagen) following the manufacturer’s protocol. The quality, purity and quantity of gDNA were assessed by gel electrophoresis in 1% agarose/1× TAE gels containing 1× SYBR Safe DNA Gel Stain (Life Technologies, Carlsbad, CA, USA) and a NanoDrop spectrophotometer (Thermo Scientific, Pittsburgh, PA, USA). DNA samples were diluted to 20–30 ng/µl to be used as a template in a multilocus PCR.

### 4.2. Genotyping by SSR Markers

The composition of the PCR multiplex reactions was designed to improve a previous panel of 27 SSR markers [10]. A modification of the dye-labels system [18] permitted the analysis of two additional loci developed thanks to the genome draft from radicchio [9], for a total of 29 SSR markers by using only three different PCR multiplex reactions (Table 3). The primers were combined into three different multiplex groups based on their annealing temperatures and their attitude to specifically and efficiently amplify the target microsatellite in multiplex reactions. Briefly, the amplification procedure was applied to 504 samples based on a three-primer system. This method consists of using SSR-targeting specific primer pairs, one of which is anchored in 5’ with a tail. This added sequence is complementary to one of the four universal (M13, PAN-1, PAN-2 and PAN-3) and fluorophore-labelled (6-FAM, VIC, NED and PET, respectively) primers used to discriminate the different loci during capillary electrophoresis (Table S7).

Every multilocus PCR was performed in a total volume of 20 µL containing 2X Platinum ^®^Multiplex PCR Master Mix (Thermo Scientific), 10% GC Enhancer (Thermo Scientific), 0.25 µM non-tailed primer, 0.75 µM tailed primer, 0.50 µM fluorophore-labelled primer, 20–30 ng of genomic DNA and distilled water up to volume. All amplifications were performed in a GeneAmp^®^ PCR 9700 thermal cycler (Applied Biosystems, Carlsbad, CA, USA). The following thermal conditions were adopted for reactions of multiplex 1: 5 min at 95 °C, followed by five cycles at 95 °C for 30 s and at 60 °C for 30 s, which decreased by 1 °C with each cycle, and at 72 °C for 30 s; and then 35 cycles at 95 °C for 30 s, at 56 °C for 30 s, and at 72 °C for 30 s. For multiplex 2 and 3, the annealing temperature was modified. Three cycles were undertaken at 95 °C for 30 s and at 56 °C for 30 s, which decreased by 1 °C with each cycle followed by 35 cycles at 95 °C for 30 s, at 54 °C for 30 s, and at 72 °C for 30 s. All reactions were terminated with a final extension of 30 min at 72 °C.

Finally, the quality of the PCR amplicons was checked by electrophoresis on 2% agarose/1× TAE gels containing 1× SYBR Safe DNA Gel Stain (Life Technologies). PCR products were dried at 65 °C. Capillary electrophoresis was performed in an ABI 3730 DNA Analyzer (Applied Biosystems). The SSR alleles were scored using PeakScanner 1.0 software (Applied Biosystems).

### 4.3. Genetic Structure of Populations

All of the analyses described below were performed for both the case study objects of this work with several exceptions that will be specified. Statistical analyses were performed with the GenAlEx 6.5 [19] and POPGENE software 1.32 [20]. Specifically, the mean values of observed heterozygosity (Ho = ∑ H/n) were computed and compared to the expected heterozygosity (He = 1 − ∑p_i_^2^). Moreover, the PIC for each of the 29 SSR loci, along with the Na, Ne and number of private alleles, was also computed. Among private alleles, we considered only those with a frequency higher than 15% in specific hybrids or populations and absent in others. Finally, marker allele and genotype frequencies for each locus were also determined.

For the comparison of 3 different years of seed production of an F1 hybrid variety, allele frequencies at each locus were also used to hypothesise the putative parental genotypes at the relative loci (e.g., A_1_A_1_×A_1_A_1_, A_1_A_1_×A_1_A_2_, A_1_A_1_×A_2_A_2_/A_1_A_2_×A_1_A_2_, A_1_A_1_×A_2_A_3_/A_1_A_2_×A_1_A_3 _and A_1_A_2_×A_3_A_4_). Moreover, the observed allele frequencies for each locus and year were matched to the most representative hypothetical profiles, and a bar chart showing the locus number of correspondences (in percentage) was drawn. Frequencies under 3% were considered deriving from external contaminations.

A UPGMA dendrogram was constructed on the base of the genetic similarity matrix, whose mean coefficients were computed between all possible pairwise combinations of the individuals belonging to the core collection. Centroids were plotted to graphically represent the genetic similarity calculated with Rohlf’s coefficient, which was calculated with the following formula GSij = m/(m + n). All genetic similarity analyses were conducted using the NTSYS software package v.2.21c [21].

The population structure of the sample collection was assessed using the clustering algorithm of STRUCTURE software [22]. All simulations were obtained by setting an admixture model without preliminary information on the population. We run a Markov Chain Monte Carlo (MCMC) model with 1,000,000 iterations and a burn-in of 200,000 samples under the assumption that the allele frequencies in the populations were correlated. Ten iterations were conducted for each value of the number of populations (K), with K ranging from 1 to 8. The method described by Evanno et al. [23] was used to evaluate the most likely estimation of K. The best value of K was calculated according to Evanno et al. [23]. Individuals showing a membership coefficient <85.0% were considered admixed.

## 5. Conclusions

The results of this study indicated that our mapped SSR marker-based method developed for genotyping analyses is reliable and informative when applied to the characterisation of the genetic structure of different cultivated populations of radicchio. The comparison among eight different subgroups of plant materials, including two OPs, four F1s and two F2s, allowed us to discover fixed marker alleles and genotypes, which is potentially useful information for assessing the genetic identity of varietal seed lots and for protecting the legal rights of breeders. The comparative analysis of different representative commercial populations also verified that OP synthetics are characterised by lower similarity and heterozygosity than F1 hybrids (which can widely vary for these two parameters) and that F2 progenies usually show intermediate mean estimates of genetic uniformity and diversity. Moreover, from the analyses of 3 production years of an F1 hybrid variety, the same set of SSR markers highlighted significant differences among the commercial seed productions. This finding was observed due to the multi-allelic nature of the derived SSR genotypes and the high PIC values found for this set of marker loci. Furthermore, our panel of SSR markers was shown to be highly informative when exploited to test the stability of an F1 hybrid variety over different production years. Overall, this study provides a cost-efficient method to genotype the Red of Chioggia biotype at different levels of hybrid varieties development, ranging from the pre-selection of parental plants to the post-production verification of the obtained seeds, as well as for the identification of seed and plant lots to prevent potential frauds. In particular, this work supports the exploitation of highly discriminant SSR markers to test whether a new variety possesses uniformity and distinctiveness traits and so can legally gain access to registration and commercialisation. In fact, to be registered and released as new plant variety, some rigorous and specific requirements concerning the distinctness (D), uniformity (U), and stability (S) of the newly bred cultivar need to be satisfied. Although most of the existing methods exploited for DUS testing are expensive and time consuming, in this work, we demonstrated, through two different case studies, that SSR markers can be adopted as a complementary tool for morphological descriptors in DUS tests.

## Figures and Tables

**Table 1:** Plant materials information, including accession IDs, number of individuals per population, population type and varietal cycles in days (d), are reported.

Accession ID	No. of Individuals	Population Type	Varietal Cycles (d)
OP-1	30	OP	70
OP-2	30	OP	110
F1-A	30	F1	70
F1-B	30	F1	110
F1-C	18	F1	100
F1-D	18	F1	70
F2-C	30	F2	100
F2-D	30	F2	70

**Table 2:** Number of alleles found across populations for each F1 hybrid, F2 population and OP synthetic. In particular, statistics refer to the mean number of alleles (Na) and number of expected alleles (Ne).

ID	OPs		F1s		F2s	
	Na	Ne	Na	Ne	Na	Ne
Mean	4.5	2.6	2.3	1.8	2.8	1.9
St. Dev.	2.4	1.2	0.9	0.6	0.9	0.5

**Table 3:** Sequences of the primer pairs used to amplify the SSR molecular markers. For each primer pair, ID, SSR linkage group (LG), motif, multiplex to which the SSR marker locus belongs, and tailed primers used (PAN1, PAN2, PAN3 or M13) are reported. All the microsatellite used in this study derive from Ghedina et al. [10], except for the two underlined SSR loci, which were newly introduced.

ID	LG	Motif		Primer Sequence and Tail	Multiplex
M1.1	1	(GA)_40_	F	[PAN3]CCAACGGATACCAAGGTGTT	1
			R	AACCGCACGGGTTCTATG	
M2.4	2	(GA)_25_	F	[M13]CCGCTCTCTCATCACTCCTC	1
			R	GCTCGAAAATCGGCTACAAC	
M2.5	2	(CT)_5_CC(CT)_13 _TT(CT)_5_	F	[PAN1]GTGCCGGTCTTCAGGTTACA	1
			R	CGCCTACCGATTACGATTGA	
M3.7	3	(CT)_22_	F	TTCGAGTCTTGCCTTAATTGTT	1
			R	[PAN1]CAGACGACCTTACGGCAACT	
M4.10a	4	(CT)_22_	F	[PAN2]CATCACCTTCACGAAAAGCA	1
			R	CGAAGACCATCCATCACCA	
M4.10b	4	(CT)_21_CATA(CA)_5_CT(CA)_5_	F	[M13] CCATTATTGGGCAGCA	1
			R	CACCAACGAACTCCTTACAAAG	
M4.11a	4	(CT)_12_N_5_(CA)_11_	F	[PAN3] GAAGGAACCTATGAACCAACCACTCA	1
			R	GTTTTGAGCCTGAGCCAGA	
M5.15	5	(CT)_11_N_7_(CAA)_5_	F	AGCACGACTCTGCTGTCTTTT	1
			R	[PAN1]CGAGCCATGTTAGGGTTTGT	
M8.22	8	(CA)_5_AA(CA)_9_	F	[PAN1]TCGTCATCAGAAACAAAGCAA	1
			R	CAAAGAAGGCACTCTTGTCG	
M9.26	9	(GATA)_3_N_19_(GA)_9_	F	[PAN2]CCTACACTCGGCCACCTACT	1
			R	TCGACGGTATAACAACACCTG	
M3.8	3	(CT)_16_	F	[PAN1]AGGAAGCGGTGTCATCTGT	2
			R	CGCCCACATATTCATTCTCA	
M6.16	6	(CT)_12_TT(CT)_15_TT(CT)_2_TT(CT)_4_	F	[PAN1]TATTGCATTGTTGTTCCTTG	2
			R	TATTTAGAAGAGGGAAATAGATG	
M7.19	7	(CT)_18_	F	ATGTCGGAGCAAAATCGTTC	2
			R	[PAN1]CATGTTCCCGCTCATGAATA	
M1.2	7	(CT)_19_	F	CCGGCAGAATTTTTAGGG	2
			R	[PAN3]CAGGTCATAGGTCCATGTGAAA	
M1.3	1	(CT)_17_	F	[PAN3]TGGAGAAAAATGAAGCAC	2
			R	GAATGAGTGAGAGAATGATAGGG	
M5.13	5	(CT)_23_	F	[M13]AGGCATAAAGAGGTGTGG	2
			R	TCAAACATGAAAACCGCTC	
M6.17	6	(CA)_8_(CT)_18_	F	CGTGTCCAAACGCAAACATTAT	2
			R	[PAN2]GCACAATTTTCCTACCACTTATCC	
M5.14	5	(TC)_11_	F	[M13]AAAGTCACACATCGCATTTCCT	2
			R	GTAGCAGCAGCAGCCATCTT	
M4.11b	4	(TG)_5_CG(TG)_7_	F	[M13]GCCATTCCTTTCAAGAGCAG	2
			R	AACCCAAAACCGCAACAATA	
M4.12	4	(CT)_8_TT(CT)_5_CC(CT)_3_TT(CT)_7_	F	GGCATCGGGATAGAAAAACA	2
			R	[PAN2]TCAATGCCTCAACAGAAATCC	
M3.9	3	(CA)_12_	F	CTGCTATGGACAGTTCCAGT	3
			R	[PAN3]CAATTCAGTTGTGATAGACGC	
M7.20	7	(CT)_31_	F	[PAN2]ACACTCACTCACACTCCGTAA	3
			R	GTCATGATGGCGTAAAAGTC	
M8.23	8	(CA)_11_(CT)_9_	F	TGTAGACACACAAAATGCACA	3
			R	[M13]ACCGGTTGAAAACATGAAAT	
M8.24	8	(TC)_16_(CA)_13_	F	[PAN2]GGTCCGTAGACTGCAGACTTTT	3
			R	CACCGTCCCACTTTTTAGG	
M9.25	9	(CA)_11_	F	[M13]GTGTGGGTGTTTGAAGAGC	3
			R	TCAAGAACATCAACGCGTAA	
M7.21	1	(CT)_13_	F	GGACACCGAGCTGGAGAA	3
			R	[PAN1]TTCCACTTTCGGGAGTTACC	
M9.27	9	(GA)_10_TAAA(GA)_5_	F	GCTAAAAGAAGTGCAAGGAGA	3
			R	[PAN1]TGTTCTTTCAAGTGCCAA	
M6.18	6	(CT)_16_	F	[PAN3]CTCAACGAATGCTTTGGACA	3
			R	CCTCGCGGTAGCTTATTGTT	
M2.6	2	(CT)_26_	F	GGAGCAGGTAGAGTCCCATC	3
			R	[PAN1]CGTTTGAAAATTTATACCAAAATG	

## Supplementary Materials

The following are available online at https://www.mdpi.com/2223-7747/8/7/213/s1, Figure S1: Pairwise genetic similarity matrix of the eight analysed populations (in percentage) based on Rohlf’s genetic similarity coefficient. The high genetic similarity values are labelled in red and the low values are labelled in green. Intermediate values are coloured in scale. Figure S2: UPGMA tree of the 216 samples analysed. The dendrogram was computed using the symmetrical genetic similarity matrix of all pair-wise comparisons among samples. Table S1: Number of alleles and PIC found across populations and loci for each of the F1 hybrids, F2 progenies and OP synthetics. In particular, statistics refer to the mean number of alleles (Na) and number of effective alleles (Ne) for each locus, population and type of population and for the whole population. Moreover, PIC values for each locus of each population are presented. Table S2: Frequency of private alleles and alleles size for each locus of the populations with frequencies ≥15% and population specific. Table S3: The sizes and the frequencies of the most frequent alleles for each locus of all the varieties and F2 progenies are reported. Table S4: The sizes of the most frequent genotype, frequency (Freq) and observed heterozygosity (Ho) for each locus of all the varieties and F2 progenies are reported. Table S5: The mean degree of expected and observed heterozygosity (He ± Std.Dev. and Ho ± Std.Dev.) for each ID: F1 hybrids, F2 progenies and OPs, singularly and by category (*). Table S6: (a) Marker loci showing allele frequencies of the private alleles only present in year 2016 (italic-bold), compared to the 2 previous production years of the hybrid variety analysed. (b) Marker loci that show alleles with meaningful differential frequencies (italic-bold) among the 3 years. Table S7: SSR primer tail and dye. List of the primer tails used with their sequence and corresponding dye.
