# Supplementary material for: Genetic Structure of Cultivated Varieties of Radicchio (Cichorium intybus L.): A Comparison between F1 Hybrids and Synthetics
Source: Plants (Basel). 2019 Jul 10;8(7):213. doi: 10.3390/plants8070213 (PMC6681312; doi:10.3390/plants8070213)
Supplement: Supplementary file 1 [file plants-08-00213-s001.pdf]

	OP-1	OP-2	F1-A	F1-B	F1-C	F1-D	F2-C	F2-D
OP-1	84.1 ± 2.6							
OP-2	78.5 ± 2.4	86.1 ± 7.1						
F1-A	78.8 ± 2.8	84.4 ± 2.0	95.0 ± 1.8					
F1-B	76.4 ± 2.2	83.7 ± 1.7	87.2 ± 1.6	95.1 ± 1.5				
F1-C	78.6 ± 2.5	78.5 ± 1.8	79.5 ± 1.5	77.4 ± 1.6	95.6 ± 1.9			
F1-D	76.9 ± 2.4	75.6 ± 2.2	76.8 ± 1.4	76.4 ± 1.5	82.5 ± 2.2	93.1 ± 2.3		
F2-C	78.8 ± 2.4	78.8 ± 2.3	80.3 ± 1.8	78.1 ± 1.7	90.2 ± 2.5	81.2 ± 2.6	88.9 ± 2.5	
F2-D	77.7 ± 2.3	76.3 ± 2.1	77.4 ± 1.9	76.9 ± 1.6	81.4 ± 1.9	90.3 ± 2.5	80.2 ± 2.6	91.1 ± 2.3

Figure S1. Pairwise genetic similarity matrix of the eight analysed populations (in percentage) based on Rohlf's genetic similarity coefficient. The high genetic similarity values are labelled in red and the low values are labelled in green. Intermediate values are coloured in scale.

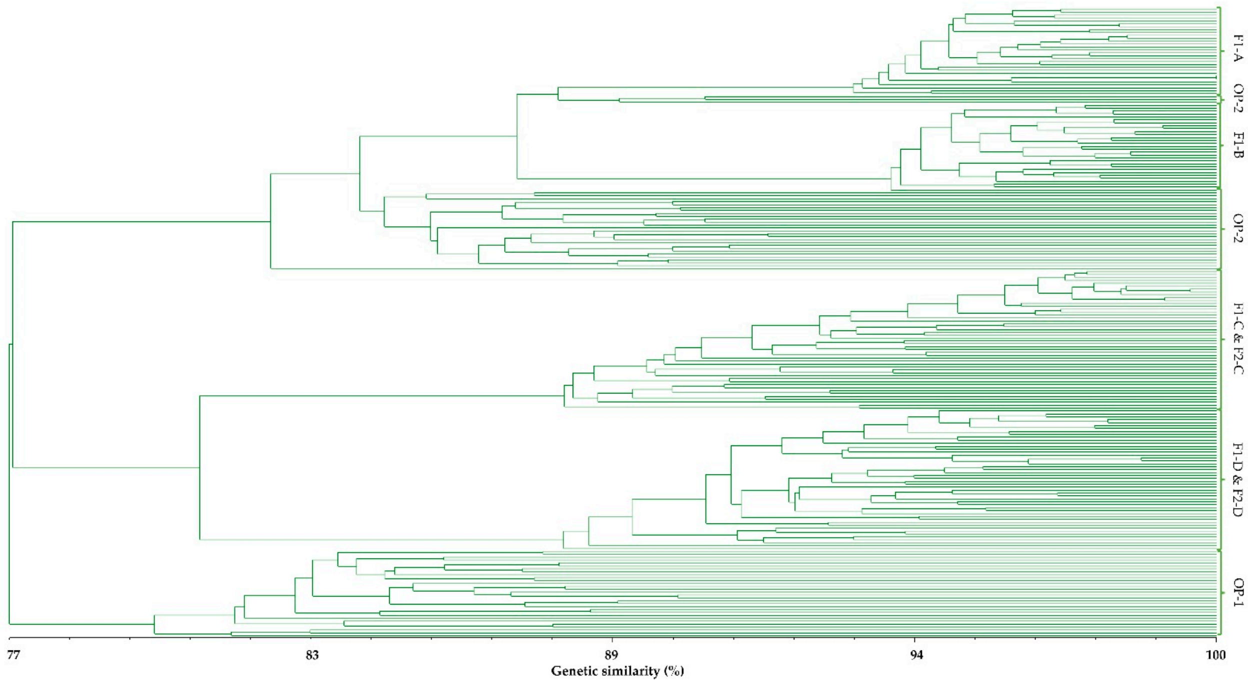

Figure S2. UPGMA tree of the 216 samples analysed. The dendrogram was computed using the symmetrical genetic similarity matrix of all pair-wise comparisons among samples.

Table S1. Number of alleles and PIC found across populations and loci for each of the F1 hybrids, F2 progenies and OP synthetics. In particular, statistics refer to the mean number of alleles (Na) and number of effective alleles (Ne) for each locus, population and type of population and for the whole population. Moreover, PIC values for each locus of each population are presented.

ID	Mean Na/Locus	Mean Ne/Locus	PIC	OP-1		OP-2		F1-A		F1-B		F1-C		F1-D		F2-C		F2-D	
Locus				Na	Ne	Na	Ne	Na	Ne	Na	Ne	Na	Ne	Na	Ne	Na	Ne	Na	Ne
M 2.4	3.1	2.2	0.7	5.0	3.1	8.0	5.1	2.0	1.0	2.0	2.0	2.0	1.1	2.0	2.0	4.0	1.7	2.0	2.0
M 4.10b	2.4	1.8	0.6	5.0	3.2	3.0	1.3	1.0	1.0	1.0	1.0	3.0	1.6	4.0	2.5	3.0	2.5	2.0	1.6
M 3.7	1.1	1.1	0.1	1.0	1.0	1.0	1.0	2.0	1.1	1.0	1.0	3.0	1.4	1.0	1.0	2.0	1.3	1.0	1.0
M 8.22	1.0	1.1	0.0	2.0	1.4	1.0	1.0	1.0	1.0	1.0	1.0	1.0	1.0	1.0	1.0	1.0	1.0	1.0	1.0
M 2.5	2.6	2.1	0.8	4.0	2.9	5.0	3.0	2.0	2.0	2.0	1.9	1.0	1.0	2.0	2.0	4.0	2.1	2.0	2.0
M 5.15	2.4	1.5	0.3	5.0	3.0	3.0	1.2	1.0	1.0	1.0	1.0	1.0	1.0	3.0	1.7	5.0	2.3	1.0	1.0
M 4.11a	3.1	2.1	0.7	4.0	1.8	6.0	1.7	2.0	1.7	2.0	1.7	3.0	2.6	3.0	2.3	4.0	2.0	4.0	2.6
M 1.1	3.9	2.3	0.8	9.0	3.4	7.0	3.1	2.0	1.8	1.0	1.0	2.0	2.0	3.0	2.1	4.0	2.8	3.0	2.1
M 9.26	3.0	2.0	0.6	6.0	2.3	3.0	1.9	2.0	1.0	2.0	1.9	3.0	2.1	4.0	3.2	4.0	2.3	3.0	1.5
M 4.10a	1.6	1.7	0.5	2.0	1.7	2.0	1.4	1.0	1.0	2.0	1.9	2.0	2.0	2.0	1.9	2.0	2.0	2.0	1.9
M 4.11b	2.0	2.1	0.5	3.0	2.6	3.0	2.1	2.0	2.0	2.0	2.0	2.0	2.0	2.0	2.0	2.0	2.0	2.0	2.0
M 5.14	1.1	1.1	0.1	2.0	1.8	1.0	1.0	1.0	1.0	1.0	1.0	1.0	1.0	1.0	1.0	2.0	1.0	1.0	1.0
M 5.13	2.1	2.1	0.7	4.0	3.0	2.0	2.0	2.0	2.0	1.0	1.0	2.0	2.0	2.0	2.0	4.0	2.9	2.0	2.0
M 6.16	2.9	1.8	0.6	6.0	2.6	4.0	2.4	2.0	1.2	3.0	1.7	2.0	2.0	2.0	1.2	4.0	2.2	2.0	1.4
M 7.19	2.4	2.0	0.6	2.0	1.9	5.0	2.5	3.0	1.7	1.0	1.0	3.0	2.5	2.0	2.0	4.0	2.2	2.0	2.0
M 3.8	3.5	2.1	0.7	4.0	1.6	8.0	4.3	2.0	1.1	2.0	1.0	1.0	1.0	4.0	3.3	4.0	1.3	4.0	3.3
M 4.12	3.9	2.5	0.8	7.0	4.1	9.0	6.1	3.0	1.3	4.0	2.7	1.0	1.0	2.0	1.3	2.0	1.8	4.0	1.4
M 6.17	2.9	2.3	0.7	2.0	2.0	8.0	4.0	2.0	1.1	4.0	3.0	4.0	3.3	2.0	1.1	2.0	1.7	3.0	2.5
M 1.2	3.1	2.1	0.8	6.0	2.5	6.0	3.1	3.0	1.6	2.0	1.2	3.0	2.1	2.0	1.9	4.0	2.6	2.0	1.5
M 1.3	3.1	2.2	0.6	4.0	1.7	7.0	4.1	4.0	2.1	2.0	2.0	2.0	2.0	2.0	2.0	4.0	2.2	2.0	2.0
M 9.25	2.5	2.1	0.7	4.0	2.4	3.0	2.1	4.0	2.7	3.0	1.7	3.0	2.1	2.0	1.5	2.0	2.0	2.0	2.0
M 8.23	2.6	1.8	0.7	3.0	2.7	3.0	1.5	4.0	1.6	2.0	2.0	2.0	1.1	3.0	2.1	3.0	1.3	3.0	1.9
M 2.6	3.9	3.0	0.8	10.0	5.2	7.0	4.8	3.0	2.4	2.0	2.0	3.0	2.3	3.0	2.1	4.0	2.9	2.0	2.0
M 7.21	1.9	1.7	0.5	1.0	1.0	2.0	2.0	2.0	1.8	2.0	2.0	2.0	1.7	2.0	2.0	3.0	1.2	3.0	2.1
M 9.27	3.4	2.4	0.8	7.0	4.2	5.0	2.6	4.0	3.9	3.0	2.3	3.0	1.5	2.0	1.5	4.0	1.3	2.0	2.0
M 7.2	2.5	2.2	0.7	6.0	4.3	1.0	1.0	2.0	1.9	3.0	2.1	2.0	2.0	3.0	2.1	3.0	2.1	2.0	1.9

M 8.24	3.3	2.5	0.8	6.0	3.9	4.0	2.0	2.0	1.3	3.0	2.6	4.0	2.2	4.0	3.1	3.0	2.4	4.0	2.1
M 6.18	3.1	2.2	0.7	6.0	4.6	4.0	1.4	1.0	1.0	3.0	2.6	2.0	1.1	4.0	3.1	4.0	1.5	3.0	2.1
M 3.9	3.5	2.6	0.8	8.0	4.0	7.0	3.2	2.0	2.0	2.0	2.0	3.0	2.5	3.0	2.5	3.0	2.7	3.0	2.1
**Mean/ Pop**	2.7	2.0	0.6	4.6	2.8	4.4	2.5	2.2	1.6	2.1	1.7	2.3	1.8	2.5	2.0	3.2	2.0	2.4	1.9

Table S2. Frequency of private alleles and alleles size for each locus of the populations with frequencies ≥15% and population specific.

ID	Locus	Allele Size (bp)	Freq
OP-1	M8.22	204	16.1%
	M4.11b	196	16.7%
	M6.17	254	50.0%
	M1.3	256	16.7%
	M9.27	278	28.3%
	M 7.2	184	22.9%
	M 6.18	173	19.0%
		175	31.0%
OP-2	M2.4	172	15.0%
	M3.8	171	20.0%
F1-B	M2.4	139	50.0%
	M1.1	247	40.0%
	M4.12	240	15.0%
	M6.17	274	15.0%
F1-C	M6.17	298	25.0%

Table S3. The sizes and the frequencies of the most frequent alleles for each locus of all the varieties and F2 progenies are reported.

ID	OP-1		OP-2		F1-A		F1-B		F1-C		F1-D		F2-C		F2-D	
**LOCUS**	**Allele size**	**Freq**	**Allele size**	**Freq**	**Allele size**	**Freq**	**Allele size**	**Freq**	**Allele size**	**Freq**	**Allele size**	**Freq**	**Allele size**	**Freq**	**Allele size**	**Freq**
**M2.4**	166	22.0%	160 - 164 - 174	< 5.0 %	170	98.0%	139	50.0%	170	97.0%	166	47.0%	170	75.0%	166	58.0%
	168	43.0%	162 - 170 - 172	< 16.0 %							170	53.0%	164	12.0%	170	42.0%
	170	30.0%	166	20.0%	168	2.0%	166	50.0%	166	3.0%			168	10.0%		
	158 - 164	< 3.0 %	168	32.0%									166	3.0%		
**M4.10b**	253 - 257	> 30.0 %							271	78.0%	255	50.0%	271	48.0%		
	255	12.0%	255	86.5%	255	100.0%	255	100.0%	255	19.0%	271	38.0%	255	40.0%	255	75.0%
	259	4.0%	257	9.6%					253	3.0%	252	6.0%	252	13.0%	271	25.0%
	271	8.0%	253	3.8%							259	6.0%				
**M3.7**					162	93.0%			162	83.0%			162	87.0%		
	162	100.0%	162	100.0%	164	7.0%	162	100.0%	164	11.0%	162	100.0%	164	13.0%	162	100.0%
									157	6.0%						
**M8.22**	201	84.0%	201	100.0%	201	100.0%	201	100.0%	201	100.0%	201	100.0%	201	100.0%	201	100.0%
	204	16.0%														
**M2.5**	215	40.0%	215	47.0%	215	57.0%	215	62.0%	225	100.0%	219	47.0%	225	67.0%	219	47.0%
	219	42.0%	219 - 225	10.0%	221	43.0%	221	38.0%			221	53.0%	221	15.0%	221	53.0%
	225	10.0%	221	30.0%									215	12.0%	215	2.0%
	221	8.0%	223	3.0%									219	7.0%		
**M5.15**	257 - 271	40.0%	271	91.7%	271	100.0%	271	100.0%	271	100.0%	255	22.0%	271	60.0%	271	100.0%
	272	6.7%	272	6.7%							271	72.0%	265	24.0%		
	259	10.0%	269	1.7%							257	6.0%	255	12.0%		
	265	3.3%											261	2.0%		
**M4.11a**	187	58.3%	187	66.7%					191	61.0%	185	39.0%	191	57.0%	185	79.0%
	185	30.0%	185	28.3%	185	98.0%	185	60.0%	185	33.0%	191	36.0%	185	32.0%	183	16.0%
	172 - 183	3.3%	183	5.0%	187	2.0%	187	40.0%	187	6.0%	187	17.0%	187	7.0%	187	5.0%
	177	1.7%									183	8.0%	183	5.0%		
**M1.1**	253	70.0%	253	16.0%	255	100.0%	247	40.0%	253	50.0%	253	41.0%	253	57.0%	253	41.0%
	255	30.0%	255	84.0%			255	60.0%	255	50.0%	255	59.0%	255	43.0%	255	59.0%
**M9.26**	130	73.0%	130	75.0%	140	70.0%	140	72.0%	140	50.0%	130	33.0%	140	60.0%	140	55.0%
	132	17.0%	162	13.0%	162	30.0%	162	28.0%	130	31.0%	140	56.0%	130	37.0%	130	24.0%
	128 - 134	< 7.0 %	134 - 140 - 150 - 156	< 5.0 %					128	19.0%	132	11.0%	128 - 134	2.0%	132 - 143	10.0%
**M4.10a**	286	46.7%	247	51.7%	247	69.0%			249	50.0%	261	47.0%	249 - 265	37.0%	261	50.0%
	247 - 249 - 265 - 272	7.0 % -5.0 %	265	15.0%	265	31.0%	247	100.0%	265	50.0%	265	50.0%	286	12.0%	265	48.0%
	261 - 267 - 273 - 284	< 5.0 %	267 - 273	10.0 % - 11.0 %							267	3.0%	247	8.0%	270	2.0%
			249 - 255 - 269	< 0.08												
**M4.11b**	187	58.0%	187	67.0%	199	48.0%	199	50.0%	199	50.0%	199	50.0%	199	50.0%	199	50.0%
	185	30.0%	185	28.0%			203	50.0%								
	172 - 183	3.0%	183	5.0%	203	52.0%			203	50.0%	203	50.0%	203	50.0%	203	50.0%
	177	17.0%														
**M5.14**	217	30.0%	219	100.0%	219	100.0%	219	100.0%	219	100.0%	219	100.0%	219	98.0%	219	100.0%
	219	70.0%											217	2.0%		
**M5.13**	243	40.0%			243	55.0%	243	100.0%	245	50.0%	245	53.0%	245	43.0%	245	53.0%

	245<br>247	40.0%<br>20.0%	245<br>243	57.0%<br>43.0%	245	45.0%			247	50.0%	247	47.0%	247<br>243<br>241	31.0%<br>24.0%<br>2.0%	247<br>241	47.0%<br>2.0%
**M6.16**	238<br>246<br>268 - 270	60.0%<br>30.0%<br>10.0%	268<br>266<br>238 - 270	60.0%<br>30.0%<br>10.0%	268<br>270<br>265	75.0%<br>18.0%<br>7.0%	268<br>265<br>244	75.0%<br>20.0%<br>5.0%	238<br>268	53.0%<br>47.0%	238<br>268	91.0%<br>9.0%	238<br>268<br>228 - 248	57.0%<br>31.0%<br>6.0%	238<br>268	81.0%<br>19.0%
**M7.19**	195<br>203	60.0%<br>40.0%	195<br>199<br>203	47.0%<br>7.0%<br>42.0%	195<br>203<br>193	71.0%<br>28.0%<br>2.0%	195	100.0%	195<br>203<br>201	53.0%<br>33.0%<br>14.0%	195<br>199	47.0%<br>53.0%	195<br>203<br>201<br>197	50.0%<br>45.0%<br>3.0%<br>2.0%	195<br>199<br>197	52.0%<br>47.0%<br>2.0%
**M3.8**	169<br>181	20.0%<br>80.0%	178<br>171<br>169 - 181 - 183 - 189	40.0%<br>20.0%<br>< 20.0 %	169<br>189	93.0%<br>7.0%	169<br>189	98.0%<br>2.0%	181	100.0%	181<br>185<br>183<br>178	36.0%<br>36.0%<br>19.0%<br>8.0%	181<br>187<br>183	88.0%<br>5.0%<br>5.0%	181<br>185<br>178<br>183	34.0%<br>34.0%<br>24.0%<br>7.0%
**M4.12**	224<br>226<br>220<br>214 - 222	40.0%<br>30.0%<br>20.0%<br>10.0%	226<br>232 - 234<br>220 - 224 - 228 -230	30.0%<br>> 10.0 %<br>< 10.0 %	226<br>228<br>224	87.0%<br>12.0%<br>2.0%	234<br>226<br>238<br>222	52.0%<br>27.0%<br>15.0%<br>7.0%	228	100.0%	224<br>216	86.0%<br>14.0%	228<br>226	68.0%<br>32.0%	224<br>216<br>219 - 225	84.0%<br>9.0%<br>4.0%
**M6.17**	254<br>264	50.0%<br>50.0%	264<br>266<br>284	38.0%<br>12.0%<br>8.0%	264<br>284	98.0%<br>3.0%	266<br>288<br>274<br>284	50.0%<br>18.0%<br>15.0%<br>18.0%	298<br>304<br>264<br>296	25.0%<br>44.0%<br>19.0%<br>13.0%	264<br>304	94.0%<br>6.0%	264<br>304	69.0%<br>31.0%	264<br>304<br>284	44.0%<br>44.0%<br>13.0%
**M1.2**	201<br>206<br>213	50.0%<br>10.0%<br>40.0%	201<br>206<br>211<br>213	7.0%<br>21.0%<br>48.0%<br>19.0%	210<br>206<br>215	75.0%<br>23.0%<br>2.0%	210	91.0%	201<br>213<br>210	56.0%<br>42.0%<br>3.0%	195<br>201	36.0%<br>64.0%	213<br>201<br>206<br>199	50.0%<br>35.0%<br>10.0%<br>5.0%	201<br>195	80.0%<br>20.0%
**M1.3**	244<br>250<br>252<br>256	75.0%<br>7.0%<br>2.0%<br>17.0%	250<br>232<br>244<br>246<br>242 - 248 - 252	31.0%<br>22.0%<br>29.0%<br>10.0%<br>< 3.0 %	244<br>232<br>227	63.0%<br>25.0%<br>4.0%	244<br>250	55.0%<br>45.0%	244<br>250	50.0%<br>50.0%	242<br>244	53.0%<br>47.0%	244<br>250<br>248<br>242	58.0%<br>35.0%<br>2.0%<br>5.0%	242<br>244	52.0%<br>48.0%
**M9.25**	177<br>195<br>199<br>183	57.0%<br>27.0%<br>13.0%<br>3.0%	175<br>177<br>183	3.0%<br>48.0%<br>48.0%	177<br>195<br>183<br>187	34.0%<br>47.0%<br>17.0%<br>2.0%	177<br>183<br>193	73.0%<br>25.0%<br>2.0%	169<br>183<br>177	47.0%<br>50.0%<br>3.0%	175<br>177	22.0%<br>78.0%	169<br>183	50.0%<br>50.0%	175<br>177	53.0%<br>47.0%
**M8.23**	231<br>229<br>221	50.0%<br>27.0%<br>23.0%	221<br>231<br>229	81.0%<br>14.0%<br>5.0%	221<br>223<br>229<br>243	78.0%<br>14.0%<br>5.0%<br>3.0%	221<br>229	50.0%<br>50.0%	229<br>231	97.0%<br>3.0%	229<br>247<br>243	53.0%<br>44.0%<br>3.0%	229<br>231	87.0%<br>13.0%	229<br>247<br>243	62.0%<br>36.0%<br>2.0%
**M2.6**	187<br>211<br>183 - 185 - 193 - 197 - 199<br>175 - 205 - 213	35.0%<br>13.0%<br>5.0 %- 1.0 %<br>< 7.0 %	193<br>197 - 205<br>195 - 213<br>187 - 199	33.0%<br>17.0 % - 18.0 %<br>12.0 %- 13.0 %<br>< 5.0 %	193<br>187<br>205	50.0%<br>40.0%<br>10.0%	193<br>205	50.0%<br>50.0%	193<br>197<br>207	44.0%<br>47.0%<br>8.0%	197<br>201<br>203	53.0%<br>44.0%<br>3.0%	207<br>197<br>193<br>199	42.0%<br>33.0%<br>20.0%<br>5.0%	201<br>197	57.0%<br>43.0%
**M7.21**	247	100.0%	237<br>247	47.0%<br>53.0%	237<br>247	31.0%<br>69.0%	237<br>247	50.0%<br>50.0%	247<br>266	72.0%<br>28.0%	237<br>247	47.0%<br>53.0%	247<br>237 - 266	90.0%<br>5.0%	237<br>247	54.0%<br>46.0%
**M9.27**	264	59.0%	306	32.0%	266	36.0%	264	59.0%	301	81.0%	301	81.0%	301	87.0%	301	57.0%

	303	22.0%	266 -278	21.0 % - 28.0 %	301	25.0%	303	22.0%	299	17.0%	303	19.0%	303	7.0%	303	43.0%
	301	19.0%	262 - 301 - 303 - 308	< 8.0 %	264	21.0%	301	19.0%	303	3.0%			297	5.0%		
					305	14.0%							299	2.0%		
	175	33.0%			132	38.0%	167	60.0%	132	58.0%	132	50.0%	132	60.0%	132	63.0%
**M7.2**	167 - 184	23.0%	167	100.0%	167	62.0%	132	35.0%	175	42.0%	175	47.0%	175	33.0%	175	37.0%
	163 - 165 - 173	< 8.0 %					169	5.0%			162	3.0%	167	7.0%		
	242	35.0%	242	67.0%			250	50.0%	242	47.0%	267	39.0%	242	52.0%	267	50.0%
**M8.24**	250 - 268	20.0 %- 28.0 %	252	20.0%	242	86.0%	267	30.0%	267	47.0%	273	33.0%	267	38.0%	273	47.0%
	270	11.0%	268	5.0%			242	20.0%	244	3.0%	269	25.0%	251	10.0%	242	2.0%
	232 - 252	4.0 % -2.0 %	270	8.0%					251	3.0%	271	3.0%			265	2.0%
	175	31.0%	187	83.0%			187	50.0%	191	97.0%	191	41.0%	191	81.0%	187	54.0%
**M6.18**	187	21.0%	181 - 191	7.0%	187	100.0%	191	30.0%	193	3.0%	187	31.0%	187 - 189	9.0%	189	45.0%
	173 - 179	19.0%	179	2.0%			189	20.0%			183	25.0%	193	2.0%	181	2.0%
	183 - 191	< 7.0 %									193	3.0%				
	223 - 239	32.0 %- 35.0 %	223	40.0%	223	52.0%	225	50.0%	223	36.0%	221	53.0%	223	48.0%	233	52.0%
**M3.9**	221 - 233	13.0 % - 8.0 %	239	37.0%	225	48.0%	239	50.0%	239	50.0%	239	28.0%	239	28.0%	221	47.0%
	217	5.0%	225	12.0%					221	14.0%	233	19.0%	221	23.0%	239	2.0%
	227 - 229 - 231	< 3.0 %	221 - 227 - 229 - 231	< 5.0 %												

Table S4. The sizes of the most frequent genotype, frequency (Freq) and observed heterozygosity (Ho) for each locus of all the varieties and F2 progenies are reported.

ID	OP-1			OP-2			F1-A			F1-B			F1-C			F1-D			F2-C			F2-D		
**Locus**	**Genotype**	**Freq**	**Ho Locus**	**Genotype**	**Freq**	**Ho Locus**	**Genotype**	**Freq**	**Ho Locus**	**Genotype**	**Freq**	**Ho Locus**	**Genotype**	**Freq**	**Ho Locus**	**Genotype**	**Freq**	**Ho Locus**	**Genotype**	**Freq**	**Ho Locus**	**Genotype**	**Freq**	**Ho Locus**
**M2.4**	168 - 168<br>166 - 168<br>168 - 170	26.7 %<br>26.7 %<br>23.3 %	66.7 %	163 - 168<br>170 - 170<br>166 - 170	20.0 %<br>13.3 %<br>13.3 %	80.0 %	170-170	96.7 %	3.3%	139-166	100.0%	100.0%	170-170	94.44 %	5.6%	166-170	94.4 %	94.4 %	170-170<br>170-164<br>170-168	50.0 %<br>23.3 %<br>20.0 %	50.0 %	166 - 170<br>166 - 166<br>170 - 170	50.0 %<br>33.3 %<br>16.7 %	50.0 %
**M4.10b**	257 - 257<br>253 - 253<br>255 - 255	30.0 %<br>23.3 %<br>6.7%	26.9 %	255 - 255<br>255 - 257<br>253 - 253	76.9 %<br>19.2 %<br>3.8%	19.2 %	255-255	100.0%	0.0%	255-255	100 %	0.0%	271-271	61.11 %	33.3 %	255-255	44.4 %	62.5 %	271-255<br>271-271<br>255-255	23.3 %<br>20.0 %<br>20.0 %	45.8 %	271 - 271<br>251 - 271<br>251 - 251	60.0 %<br>20.0 %<br>13.3 %	21.4 %
**M3.7**	162-162	100.0%	0.0 %	162 - 162	100.0%	0.0%	162-162	93.3 %	0.0%	162-162	100 %	0.0%	162-162	100%	0.0%	162-162	100.0%	0.0%	162-162<br>164-164	86.7 %<br>13.3 %	0.0%	162-162	100.0 %	0.0%
**M8.22**	201 - 201<br>201 - 204	71.4 %<br>25.0 %	25.0 %	201 - 201	100.0%	0.0%	201-201	100.0%	0.0%	201-201	100 %	0.0%	201-201	100%	0.0%	201-201	100.0%	0.0%	201-201	100.0%	0.0%	201-201	100.0 %	0.0%

**M2.5**	215 - 221<br>219 - 219	32.1 %<br>25.0 %	56.7 %	215 - 215<br>221 - 221	32.1 %<br>25.0 %	33.3 %	215- 215<br>221- 221	46.7 %<br>33.3 %	17.2 %	215-215<br>215-221	0.5%<br>20.0 %	20.7 %	225-225	100%	0.0%	219-221	94.4 %	94.4 %	225-225<br>215-225	56.7 %<br>16.7 %	36.7 %	219 - 221<br>219 - 219	56.7 %<br>23.3 %	64.3 %
**M5.15**	257 - 257<br>271 - 271<br>259 - 271	26.7 %<br>26.7 %<br>6.7%	40.0 %	225 - 215<br>271 - 271<br>273 - 273	21.4 %<br>90.0 %<br>6.7%	3.3%	271- 271	100. 0%	0.0%	271-271	100 %	0.0%	271-271	100%	0.0%	255-271<br>271-271	33.3 %<br>50.0 %	44.4 %	271-271<br>265-265<br>255-271	53.3 %<br>20.0 %<br>10.0 %	17.2 %	271-271	100.0 %	0.0%
**M4.11a**	187 - 187<br>185 - 185	46.7 %<br>16.7 %	23.3 %	187 - 187<br>185 - 185	53.3 %<br>16.7 %	40.0 %	185- 185	96.7 %	3.3%	185-187	73.3 %	73.3 %	185-185<br>191-191	61.11 %<br>33.33 %	0.0%	185-185<br>187-191	27.8 %<br>33.3 %	72.2 %	191 - 191<br>185 - 185	40.0 %<br>26.7 %	33.3 %	185 - 185<br>183 - 183	76.7 %<br>13.3 %	3.5%
**M1.1**	253 - 253<br>255 - 255	63.3 %<br>23.3 %	53.3 %	255 - 255<br>253 - 253	82.8 %<br>13.8 %	60.0 %	255- 255	100. 0%	0.0%	247-247<br>247-255	20.0 %<br>20.0 %	40.0 %	253-255	100%	100. 0%	253-255	77.8 %	82.4 %	253 - 253<br>253 - 255	40.0 %<br>33.3 %	33.3 %	253 - 255<br>253 - 253	40.0 %<br>36.7 %	42.9 %
**M9.26**	130 - 130<br>132 - 132	66.7 %<br>10.0 %	33.3 %	130 - 130<br>130 - 162	63.0 %<br>18.5 %	26.7 %	140- 162	60.0 %	60.0 %	140-140<br>140-162	43.3 %<br>56.7 %	56.7 %	130-140<br>128-140	61.11 %<br>38.89 %	100. 0%	130-140	66.7 %	88.9 %	130 - 140<br>140 - 140	66.7 %<br>23.3 %	80.0 %	130 - 140<br>132 - 144	46.7 %<br>20.0 %	69.0 %
**M4.10a**	273 - 286<br>286 - 286<br>273 - 273	26.7 %<br>26.7 %<br>6.7%	13.3 %	247 - 247<br>247 - 265<br>247 - 267	33.3 %<br>13.3 %<br>13.3 %	3.5%	247- 265	60.0 %	62.1 %	247-247	100. 0%	0.0%	249-265	88.89 %	88.9 %	261-265	88.9 %	94.4 %	249 - 265<br>265 - 265<br>249 - 249	20.0 %<br>13.3 %<br>10.0 %	76.7 %	261 - 265<br>261 - 261	40.0 %<br>26.7 %	44.8 %
**M4.11b**	199 - 203<br>196 - 199	60.0 %<br>33.3 %	93.3 %	199 - 203<br>199 - 199	86.7 %<br>6.7%	90.0 %	199- 203	96.7 %	96.7 %	199-203	100. 0%	100. 0%	199-203	100%	100. 0%	199-203	100. 0%	100. 0%	199-203	100. 0%	100. 0%	199-203	100.0 %	100. 0%
**M5.14**	219 - 219<br>217 - 219	56.7 %<br>20.0 %	20.7 %	219 - 219	100. 0%	0.0%	219- 219	100. 0%	0.0%	219-219	100. 0%	0.0%	219-219	100%	0.0%	219-219	100. 0%	0.0%	219-219	96.7 %	3.3%	219-219	100.0 %	0.0%
**M5.13**	243 - 247<br>243 - 245<br>243 - 243	28.0 %<br>28.0 %<br>20.0 %	63.3 %	243 - 245<br>245 - 245<br>243 - 243	60.0 %<br>26.7 %<br>13.3 %	60.0 %	243- 245	90.0 %	90.0 %	243-243	100. 0%	0.0%	245-247	100.0 0%	100. 0%	245-247	94.4 %	94.4 %	243 - 245<br>245 - 247<br>245 - 245	30.0 %<br>26.7 %<br>20.0 %	79.3 %	247 - 247<br>249 - 249<br>245 - 245	53.3 %<br>26.7 %<br>16.7 %	56.7 %
**M6.16**	238 - 238<br>246 - 246	43.3 %<br>20.0 %	27.6 %	268 - 268<br>238 - 268	33.3 %<br>16.7 %	50.0 %	268- 268	30.0 %	21.4 %	268-268	43.3 %	31.8 %	238-268	94.44 %	94.4 %	238-238	77.8 %	17.7 %	238 - 238<br>268 - 268	46.7 %<br>20.0 %	20.7 %	238-238	63.3 %	31.0 %

	238 - 246	6.7%		266 - 266	16.7%														238 - 268	16.7%				
**M7.19**	195 - 203<br>195 - 195	73.3%<br>23.3%	73.3%	195 - 203<br>195 - 195	73.3%<br>6.7%	90.0%	195-203	53.3%	58.6%	195	100.0%	0.0%	195-203	66.67%	94.4%	195-199	94.4%	94.4%	193 - 203	86.7%	96.7%	195 -199<br>195 - 195	43.3%<br>30.0%	46.7%
**M3.8**	181 - 181<br>169 - 181<br>169 - 187	90.5%<br>4.8%<br>28.6%	36.7%	178 - 178<br>171 - 171<br>171 - 181	30.0%<br>10.0%<br>10.0%	53.3%	169-169	86.7%	13.3%	169-169	93.3%	3.5%	181-181	100.00%	0.0%	181-185	61.1%	88.9%	181 - 181	76.7%	17.2%	181 - 185<br>181 - 181<br>185 - 185	30.0%<br>16.7%<br>16.7%	44.8%
**M4.12**	224 - 224<br>226 - 226	36.7%<br>23.3%	10.0%	226 - 234<br>220 - 220	13.3%<br>6.7%	60.0%	226-226	73.3%	26.7%	226-234<br>234-234	30.0%<br>33.3%	56.7%	228-228	100%	0.0%	224-224	77.8%	16.7%	228 - 228<br>226 - 228	46.7%<br>16.7%	24.0%	224 - 224<br>216 - 224	78.6%<br>10.7%	10.7%
**M6.17**	254 - 254<br>264 - 264<br>254 - 264	42.3%<br>42.3%<br>15.4%	15.4%	264 - 264<br>286 - 286<br>266 - 266	32.0%<br>24.0%<br>12.0%	12.0%	264-264	63.3%	5.0%	266-266<br>266-288	20.0%<br>20.0%	60.0%	298-304<br>264-304	22.22%<br>16.67%	87.5%	264-264	38.9%	12.5%	264 - 264<br>304 - 304<br>264 - 304	36.7%<br>13.3%<br>10.0%	16.7%	264 - 264<br>304 - 304<br>264 - 304	37.5%<br>37.5%<br>12.5%	12.5%
**M1.2**	201 - 201<br>201 - 213<br>213 - 213	32.1%<br>32.1%<br>21.4%	44.8%	211 - 211<br>206 - 206<br>213 - 213	44.8%<br>20.7%<br>17.2%	6.9%	206-210<br>210-210	43.3%<br>46.7%	50.0%	210-210	76.7%	17.9%	201-213	83.33%	88.9%	195-201	72.2%	72.2%	201 - 213<br>213 - 213<br>201 - 201	40.0%<br>23.3%<br>13.3%	56.7%	201 - 201<br>195 - 201	60.0%<br>40.0%	40.0%
**M1.3**	244 - 244<br>244 - 256<br>244 - 250	53.3%<br>30.0%<br>10.0%	46.7%	244 - 244<br>232 - 250<br>250 - 250	20.7%<br>20.7%<br>17.2%	44.8%	232-244<br>244-244	33.3%<br>36.7%	60.7%	244-244<br>244-250	33.3%<br>43.3%	43.3%	244-250	100%	100.0%	242-244	94.4%	94.4%	244 - 250<br>244 - 244	40.0%<br>33.3%	53.3%	242 - 242<br>244 - 244<br>242 - 244	36.7%<br>33.3%<br>30.0%	30.0%
**M9.25**	177 - 195<br>177 - 177<br>177 - 199	40.0%<br>30.0%<br>10.0%	66.7%	177 - 183<br>175 - 183<br>175 - 177	93.3%<br>3.3%<br>3.3%	100.0%	177-195	56.7%	89.7%	177-177<br>177-183	50.0%<br>43.3%	46.7%	169-183	94.4%	100.0%	175-177	66.7%	22.2%	169 - 183	100.0%	100.0%	175 - 177<br>175 - 175<br>177 - 177	53.3%<br>26.7%<br>20.0%	53.3%
**M8.23**	231 - 231<br>221 - 221	37.5%<br>16.7%	29.2%	169 - 169<br>169 - 183	65.5%<br>24.1%	34.5%	221-221	66.7%	17.2%	221-229	100%	100.0%	229-229	94.4%	5.6%	229-247	88.9%	94.4%	229 - 229<br>221 - 229	73.3%<br>23.3%	26.7%	229 - 247<br>229 - 229	72.4%<br>24.1%	75.9%
**M2.6**	187 - 187	20.0%	63.3%	193 - 197	13.3%	73.3%	187-193	76.7%	100.0%	193-205	100.0%	100.0%	193-197	77.8%	94.4%	197-201	88.9%	94.4%	193 - 207	40.0%	73.3%	197 - 201	53.3%	53.3%

	187 - 213<br>187 - 211	10.0 %<br>10.0 %		193 - 205<br>193 - 213	13.3 %<br>13.3 %														197 - 207<br>197 - 197	23.3 %<br>20.0 %		201 - 201<br>197 - 197	30.0 %<br>16.7 %	
**M7.21**	247 - 247	100.0%	0.0 %	237 - 247<br>247 - 247<br>237 - 237	37.9 %<br>34.5 %<br>27.6 %	37.9 %	237-247	60.0 %	62.1 %	237-247	100.0%	100.0%	247-266	55.6 %	55.6 %	237-247	94.4 %	94.4 %	247 - 247<br>247 - 266	80.0 %<br>10.0 %	20.0 %	237 - 247<br>237 - 237<br>247 - 247	40.0 %<br>33.3 %<br>23.3 %	43.3 %
**M9.27**	266 - 278<br>266 - 306<br>278 - 306	16.7 %<br>10.0 %<br>10.0 %	80.0 %	264 - 266<br>264 - 264<br>266 - 266	31.0 %<br>27.6 %<br>13.8 %	58.6 %	266-301	30.0 %	92.9 %	264-303<br>264-301	33.3 %<br>26.7 %	72.4 %	301-301	72.2 %	16.7 %	301-301<br>301-303	61.1 %<br>38.9 %	38.9 %	301 - 301	73.3 %	26.7 %	301 - 303<br>301 - 301<br>303 - 303	43.3 %<br>33.3 %<br>20.0 %	44.8 %
**M7.2**	174 - 184<br>167 - 167	26.1 %<br>21.7 %	37.5 %	167 - 167	100.0%	0.0%	132-167	70.0 %	70.0 %	132-167	66.7 %	70.0 %	132-175	83.3 %	83.3 %	132-175	94.4 %	100.0%	132 - 175<br>132 - 132	66.7 %<br>20.0 %	80.0 %	132 - 175<br>132 - 132	7333.3%<br>2666.7%	73.3 %
**M8.24**	242 - 268<br>250 - 268<br>242 - 270	22.2 %<br>18.5 %<br>11.1 %	70.4 %	242 - 242<br>252 - 270<br>252 - 252	60.0 %<br>13.3 %<br>10.0 %	26.7 %	242-242	70.0 %	27.6 %	250-267	60.0 %	100.0%	242-267	94.4 %	100.0%	269-273<br>267-273	38.9 %<br>27.8 %	83.3 %	242 - 267<br>242 - 242	40.0 %<br>26.7 %	60.0 %	269 - 273<br>269 - 269<br>273 - 273	4000.0%<br>2666.7%<br>2333.3%	48.3 %
**M6.18**	173 - 173<br>175 - 175	10.7 %<br>10.7 %	51.7 %	187 - 187<br>181 - 181	73.3 %<br>3.3%	3.7%	187-187	100.0%	0.0%	187-191	60.0 %	100.0%	191-191	94.4 %	5.6%	183-187<br>191-191	44.4 %<br>33.3 %	56.3 %	191 - 191<br>187 - 191	63.3 %<br>16.7 %	34.5 %	187-187<br>183-183	4000.0%<br>3333.0%	21.4 %
**M3.9**	223 - 239<br>239 - 239	20.0 %<br>16.7 %	70.0 %	223 - 239<br>225 - 225	73.3 %<br>6.7%	86.7 %	223-225	96.7 %	96.7 %	225-239	100.0%	100.0%	223-239	72.2 %	100.0%	221-239<br>233-239	55.6 %<br>38.9 %	94.4 %	223 - 223<br>221 - 239	40.0 %<br>40.0 %	56.7 %	221-233<br>233-233	5333.0%<br>2333.0%	56.7 %

Table S5. The mean degree of expected and observed heterozygosity (He ± Std.Dev. and Ho ± Std.Dev.) for each ID: F1 hybrids, F2 progenies and OPs, singularly and by category (*).

ID	He %	Ho %
OP-1	57 ± 0.04	43 ± 9.73
OP-2	49 ± 0.05	40 ± 8.29
F1-A	29 ± 0.40	39 ± 6.59
F1-B	35 ± 0.05	49 ± 5.23
F1-C	36 ± 0.05	53 ± 4.2
F1-D	45 ± 0.04	67 ± 11.9
F2-C	45 ± 0.03	46 ± 8.48
F2-D	42 ± 0.04	40 ± 10.19
F1 *	36 ± 6.42	52 ± 11.56
F2 *	44 ± 2.18	43 ± 4.35
OP *	53 ± 5.91	42 ± 1.75

Table S6. (**a**) Marker loci showing allele frequencies of the private alleles only present in year 2016 (italic-bold), compared to the 2 previous production years of the hybrid variety analysed. (**b**) Marker loci that show alleles with meaningful differential frequencies (italic-bold) among the 3 years.

(a)

**Locus**	**M1.2**			**M1.3**			**M2.6**			**M8.24**			**M9.25**		
**Allele\year**	**2014**	**2015**	**2016**	**2014**	**2015**	**2016**	**2014**	**2015**	**2016**	**2014**	**2015**	**2016**	**2014**	**2015**	**2016**
**A_1**	48.9%	50.0%	34.5%	26.6%	19.8%	22.8%	0.6%			99.5%	98.9%	87.4%	99.5%	100.0%	85.2%
**A_2**	51.1%	50.0%	50.0%			2.5%	30.8%	49.5%	39.0%			0.5%	0.5%		
**A_3**			***15.5%***	72.9%	79.3%	66.1%	0.6%				1.1%				***14.8%***
**A_4**				0.5%	0.9%		67.0%	50.5%	50.0%	0.5%					
**A_5**						***8.6%***	1.1%					***12.1%***			
**A_6**									***11.1%***						

(b)

**Locus**	**M8.23**			**M9.26**			**M9.27**		
**Allele\year**	**2014**	**2015**	**2016**	**2014**	**2015**	**2016**	**2014**	**2015**	**2016**
**A_1**	1.1%			***25.8%***	0.5%		30.8%	24.2%	25.0%
**A_2**	21.7%	50.0%	34.6%	48.4%	75.8%	30.3%	27.3%	49.5%	35.8%
**A_3**	0.5%			0.6%		***46.1%***		0.5%	
**A_4**	50.0%	50.0%	48.9%	24.7%	23.7%	23.6%	20.4%	25.8%	24.3%
**A_5**			1.1%	0.6%			0.6%		1.4%
**A_6**	1.6%						***19.8%***		***13.5%***
**A_7**	***24.5%***		***15.4%***				0.6%		
**A_8**	0.5%							0.6%	

Table S7. SSR primer tail and dye. List of the primer tails used with their sequence and corresponding dye.

Universal Primer	Sequence 5′–3′	Dye
M13	TTGTAAAACGACGGCCAGT	6-FAM
PAN1	GAGGTAGTTATTGTGGAGGAC	VIC
PAN2	GGAATTAACCGCTCACTAAAG	NED
PAN3	TGTAGAAAGACGAAGGGAAGG	PET
